# Spermiogram, Kinetics, Flow Cytometric Characteristics and DNA Damage Degree in Boar Ejaculates: Summarization and Clustering

**DOI:** 10.3390/vetsci11090420

**Published:** 2024-09-09

**Authors:** Raquel Ausejo-Marcos, María Teresa Tejedor, Sara Miguel-Jiménez, Belén Gómez-Giménez, Cristina Soriano-Úbeda, Noelia Mendoza, Alejandro Vicente-Carrillo, William Fernando Hurtado, Celia Ávila Holguín, Bernardino Moreno, María Victoria Falceto

**Affiliations:** 1 Department of Biotechnology R&D, Magapor S.L., 50600 Ejea de los Caballeros, Spain; sveterinarios@magapor.com (R.A.-M.); vetelab@magapor.com (N.M.); 2 Department of Anatomy, Embryology and Genetics, Centro de Investigación Biomédica en Red: Enfermedades Cardiovasculares, Faculty of Veterinary Medicine, University of Zaragoza, 50013 Zaragoza, Spain; 3 Department of Research and Development, CiencIAnova Magapor, 50018 Zaragoza, Spain; investigacion@magapor.com (S.M.-J.); alevic05@ucm.es (A.V.-C.); 4 Department of Molecular Biology (Cell Biology), University of León, 24071 León, Spain; bgomg@unileon.es; 5 INDEGSAL, University of León, 24071 León, Spain; msoru@unileon.es; 6 Department of Veterinary Medicine, Surgery, and Anatomy (Animal Medicine and Surgery), University of León, 24071 León, Spain; 7 Department of Animal Pathology, Obstetrics and Reproduction, Faculty of Veterinary Medicine, University of Zaragoza, 50013 Zaragoza, Spain; 809796@unizar.es (W.F.H.); 738714@unizar.es (C.Á.H.); bmoreno@unizar.es (B.M.); vfalceto@unizar.es (M.V.F.)

**Keywords:** boar ejaculate, semen analysis, semen classification, DNA damage

## Abstract

**Simple Summary:**

Artificial insemination is nowadays a widespread practice; boar semen analysis is an essential key to guaranteeing pregnancy and high prolificacy. This analysis can include sperm motility, concentration, morphology, membrane integrity, DNA damage and seminal plasma components; a huge amount of data is available for each ejaculate and the interpretation of these results is complicated. This study aims to summarize these data, to classify ejaculates in several categories (clusters) and to investigate the potential differences among clusters on fertility and prolificacy. Two groups of Pietrain boars were studied (90 and 30 ejaculates weekly in groups 1 and 2, respectively). Computer-assisted semen analysis (CASA) and flow cytometry analysis were performed, and statistical analysis of the results was carried out using SPSS v.26 software. In both groups, variables were grouped in three principal components related to sperm velocity, linearity and DNA damage that allowed the ejaculates to be grouped into four clusters. Although an adequate description of the characteristics of the ejaculates was achieved, no differences were found among clusters for fertility or prolificacy since, with efficient quality control of semen, no relationships with fertility parameters would be expected once the minimum requirements have been met.

**Abstract:**

Boar semen analysis includes sperm motility, concentration, morphology and other more complex analyses such as membrane integrity, DNA damage and seminal plasma components. This study aims to summarize these numerous data by linear combinations of them, to classify ejaculates in several categories (clusters) and to investigate the potential differences among clusters on fertility and prolificacy. Young Pietrain boars (23 ± 3.6 months) were investigated: ten boars from the Nucléus genetic line (group 1: 90 ejaculates weekly) and five boars from the Batallé genetic line (group 2: 30 ejaculates weekly). Computer-assisted semen analysis (CASA) examined motility. Sperm viability, acrosome reaction, early apoptosis, mitochondrial activity and DNA damage were studied by flow cytometry analysis. SPSS v.26 software was used to perform principal component analysis (PCA) and clustering. Three principal components (PC1: speed; PC2: linear path; PC3: DNA damage) were detected and four clusters identified in both groups. Clusters also differed significantly in several variables not included in these PCs (group 1: beat cross frequency and poly (ADP-ribose) polymerase; group 2: cathepsin B, abnormal forms, mitochondrial activity and high DNA stainability). PCA and clustering achieved adequate description of these ejaculates, but no differences among clusters were found for fertility or prolificacy, probably because the minimum sperm requirements had been met.

## 1. Introduction

Sperm motility is currently the most widely used parameter in many mammalian species, including boars, as a fast and accurate method to discard ejaculates with low quality [1,2]. This is done using computer-assisted semen analysis (CASA) equipment, a tool that accurately evaluates semen motility [3,4,5]. The information from CASA includes numerous variables; therefore, for a more comprehensive overview of them, several statistical techniques for resuming original variables and classification of samples have been applied [6]. Data on motility were used for detection of sperm sub-populations in donkeys and horses [7]. In pigs, recent studies refer to the usefulness of kinematics and morphological characteristics in classifying boar sperm [8,9,10]. Meanwhile, positive correlations between motility and quality variables have been found in bulls [11]. Other sperm parameters have been widely studied due to their relation to spermatozoon functionality, such as the plasma membrane and acrosome integrity, mitochondrial membrane potential, intracellular calcium concentration and content of reactive oxygen species or lipid peroxidation, using flow cytometry as one of the main tools [12].

The hypo-osmotic swelling test (HOST) and osmotic resistance test (ORT) give us an idea of the membrane integrity.

Markers of DNA damage, such as DNA fragmentation, have recently attracted much interest [13,14] and are considered necessary for objective single ejaculate evaluation [13]. PARP1, a member of the poly (ADP-ribose) polymerase (PARP) family of enzymes, plays a role in detecting and repairing DNA damage through poly (ADP-ribose) (PAR) labeling. When severe DNA damage occurs, PARP1 is cleaved into cPARP peptide by active caspases. In spermatozoa, this process is associated with chromatin remodeling during spermatogenesis and the activation of apoptotic pathways.

Seminal plasma is essential for sperm functionality and fertility, offering a nutrient-rich medium that supports sperm viability and motility. It contains ions, proteins, enzymes and antioxidants that protect sperm from oxidative stress, among other functions.

Analyzing all these components is crucial to understanding their relationship with fertility and prolificacy, providing insights into reproductive success that could help the industry in detecting differences between boars used in breeding programs. Hence, integrating all this information in an easy way is necessary to better evaluate ejaculate quality.

The first objective of the present study was to summarize the numerous characteristics usually analyzed in semen samples in a small number of variables (linear combinations of original variables, principal components) for easier description of ejaculate quality. The second objective was to classify the ejaculates into several categories (clusters) based on their quality, assessed by these linear combinations. Finally, the potential differences among these ejaculate clusters in fertility and prolificacy were investigated.

## 2. Materials and Methods

Acronyms used in this work are shown in Supplementary Materials (Table S1).

### 2.1. Animals

The animal study protocol was approved by the Ethics Committee of the University of Zaragoza (PI36/24, 25 June 2024).

A total of 15 Pietrain boars (aged 23 ± 3.6 months) from two commercial artificial insemination (AI) studs in Spain were investigated. Group 1 consisted of 10 boars from the Nucléus genetic line with weekly sperm extractions from 14 January to 11 March 2022. Group 2 consisted of 5 boars from the Batallé genetic line with sperm extractions every four days and sperm analyzed weekly from 18 March to 22 April 2022. For reasons of laboratory logistics and biosecurity (days off prior to entering the farms), we were forced to carry out the evaluations successively and not simultaneously. The consideration of different lines was due to the fact that the boar studs were working with different genetic companies but the same breed, so we did not have the option to select the same genetics. Both boar studs were light, temperature- and humidity-controlled, and located in the same geographical area, and the months of study corresponded to the increasing photoperiod so that no significant quality changes should be expected.

All boars in the study were selected according to their semen quality and health history; animals under any sanitary treatments, illness, infections or with low seminal quality were excluded. Moreover, both studs performed blood tests for notifiable diseases plus PRRS (porcine reproductive and respiratory syndrome) virus. The last of these tests was performed in every ejaculate collection. Boars were housed in individual crates of at least 6 m^2^, with free access to water. Boars were in good health and were fed once a day with a commercial boar-specific diet. The barn’s light regime and temperature were controlled.

### 2.2. Semen Collection and Preparation of Seminal Doses

(Semen) was collected using the double-gloved-hand technique and filtered to remove the gel fraction. Temperature and humidity were controlled in both boar studs; therefore, the same environmental conditions were followed throughout the year. The pre-sperm fraction was discarded. Upon entry to the AI stud laboratory, the temperature and weight of the ejaculates were recorded. Semen quality was evaluated with the AI stud CASA system, and ejaculates with <70% sperm motility and >25% abnormal sperm were excluded. Only ejaculates that met the established minimum sperm quality limits (Table 1) were used in this study. These limits were established according to Magapor SL criteria which in turn follow the recommendations from several authors [14,15] and national associations of pig breeders like the ANPS (Asociacion Nacional de Criadores de Ganado Porcino Selecto, Madrid, Spain) and BPEX (British Pig Executive, England, UK). Therefore, a maximum of 90 data and 30 data were available from seminal doses in groups 1 and 2, respectively.

Ejaculates were diluted at the AI center after evaluation to produce semen doses for delivery to sow farms. AI semen doses were prepared by diluting the ejaculates with a high-performance commercial extender (Duragen^®^; Magapor SL) to a final concentration of 2.9 × 10^7^ spermatozoa/mL. Seminal doses of 45 mL (total 1.3 × 10^9^ spermatozoa/AI dose) were packaged in blisters on the AI stud and kept at 15–17 °C until delivery to destination. Two semen doses were kept in the AI stud for 5 days to assess its conservation capacity, and the other two doses plus the remaining ejaculate were sent to an external laboratory (ciencIAnova, Magapor AIE, Zaragoza, Spain) for the assessing of sperm parameters and seminal plasma isolation. The rest of the doses were sent and used for inseminations in sow farms.

### 2.3. Seminal Plasma Analyses

Pure ejaculates were processed in the external laboratory to separate the seminal plasma (SP). After centrifugation of fresh semen (1500× *g* for 10 min), supernatant (seminal plasma, without sperm cells) was separated for analyses. The supernatant (SP free from cells) was then aliquoted and frozen at −80 °C until analysis. Pellet (centrifuged semen without seminal plasma, containing sperm cells) was not used in this study. Sperm analyses were performed in semen diluted with the extender mentioned above (Duragen^®^) just after the collection. Apart from pH, some other relevant components in boar SP were evaluated, as explained below.

Protein concentration was determined by the Micro BCA Protein Assay Kit (Thermo Fisher Scientific Inc., Waltham, MA, USA) following the manufacturer’s instructions. Fructose, zinc and citrate concentration was assessed using the commercially available D-Glucose/D-Fructose, BROMO-PAPS and Citrate/Citric Acid Kits, respectively, and the automatic A15 Access Analyzer (kits and analyzer from Biosystems, Barcelona, Spain) following manufacturer’s instructions.

Enzymatic activity was measured for the following enzymes using commercially available kits following manufacturer’s instructions: superoxide dismutase (SOD) by a colorimetric kit (Thermo Fisher Scientific Inc., Waltham, MA, USA), β-galactosidase using the Mammalian β-Galactosidase Assay Kit (Thermo Fisher Scientific), glutathione peroxidase 5 (GPX5) by the Pig Epididymal secretory GPX ELISA Kit (MyBioSource Inc., San Diego, CA, USA), cathepsin B using the Catheptsin B Activity Assay Kit and alkaline phosphatase by the Alkaline Phosphatase Assay Kit, both from Abcam (Cambridge, UK).

### 2.4. Evaluation of Seminal Doses

#### 2.4.1. Sperm Motility and Concentration

Sperm motility analysis was performed using a commercial computer-assisted sperm analysis system (CASA) specific to boars (Magavision^®^, Magapor SL, Ejea de los Caballeros, Spain) and a microscope equipped with a ×10 negative phase-contrast lens.

Materials used were pre-warmed to 36–37 °C and maintained at this temperature during the analysis by a heated slide holder. Aliquots of 1 mL of sperm samples were warmed up for 5 min at 37 °C before motility assessment. Samples (3.5 μL) were placed in a counting chamber (Magapor SL), and five different fields with at least 500 sperm cells in total were captured.

Sperm concentration and the following sperm kinematic parameters were evaluated: percentages of total sperm motility (TMot), progressive sperm motility (PMot), fast, intermediate, slow and static spermatozoa, curvilinear velocity (VCL, µm/s), straight line velocity (VSL, µm/s), average velocity path (VAP, µm/s), linearity (LIN, VSL/VCL, %), straightness (STR, VSL/VAP, %), wobble (WOB, VAP/VCL, %), amplitude of lateral head displacement (ALH, µm) and beat cross frequency (BCF, Hz).

#### 2.4.2. Sperm Morphology and Acrosome Status

Sperm morphology was assessed by examining a smear of the semen sample stained (1:1) with eosin–nigrosine solution (Magapor, Zaragoza, Spain) under a bright-field microscope at ×1000 magnification. Sperm cells displaying abnormalities in the head, midpiece or tail, or exhibiting cytoplasmic droplets (proximal, PD, and distal, DD), were categorized as abnormal. The percentage of total abnormal forms (AF) and the percentage of each specific type of abnormality were recorded.

Acrosome status was evaluated and the percentage of spermatozoa with intact acrosome was counted in the same smear used to evaluate the different abnormalities.

#### 2.4.3. Short Hypo-Osmotic Swelling Test and Short Osmotic Resistance Test

The short hypo-osmotic swelling test (sHOST) and the short osmotic resistance test (sORT) were used to evaluate the functional integrity of the sperm membrane and acrosome, providing an indication of sperm viability [16]. sHOST and sORT were performed as described previously [17]. Briefly, semen aliquots of the diluted semen (2.9 × 10^7^ spermatozoa/mL) were warmed for 5 min in a water bath at 37 °C. Then, 30 µL of each sample was added to a tube containing 100 µL of hypo-osmotic solution (75 mOsm/kg) in a water bath. Hypo-osmotic solution was prepared by serial dilutions of Beltsville Thawing Solution (BTS: 37 g dextrose hydrate, 6 g sodium citrate dihydrate, 1.25 g sodium bicarbonate, 1.25 g disodium ethylenediamine tetraacetate (EDTA) and 0.75 g potassium chloride per liter). After an incubation of 15 min in the hypo-osmotic solution, semen was fixed with 2% glutaraldehyde solution.

The percentage of viable spermatozoa (those with curled tails) and the percentage of spermatozoa with intact acrosomes (acro %) were assessed using a smear stained with eosin–nigrosine and examined under a bright-field microscope at ×1000 magnification.

#### 2.4.4. Flow Cytometry Analysis

For the assessment of sperm membrane and acrosome integrity, apoptosis and mitochondrial activity, a BD Accuri™ C6 flow cytometer with BD software (Becton Dickinson, Madrid, Spain) was used. It is equipped with 2 laser sources (blue, 488 nm and red, 640 nm) and 4 fluorescence detectors (FL1-533 nm, FL2-585 nm, FL3-670 nm, LP FL4-675 nm). A minimum of 20,000 events were recorded for each sample. The sperm population was identified and selected for further analysis based on its unique forward scatter (FS) and side scatter (SS) characteristics, while non-sperm events were excluded from the analysis.

FACS (Becton Dickinson) with CellQuest v3 software (Becton Dickinson) was used for sperm chromatin structure assay (SCSA) analysis, exciting the samples with blue laser and recording the emitted fluorescence detected with the 525/50 nm and 665/730 nm filters for the green and red fluorescence of the acridine orange, counting over 2000 sperm cells (aiming for 5000) within maximum 3 min. Consistent instrument settings were used for all samples following the procedure described by Martinez-Pastor [18].

To detect the presence of PARP1 and its derivatives, cPARP and PAR, samples were analyzed with a Cytek^®^ Aurora spectral cytometer (Cytek^®^ Biosciences, Amsterdam, The Netherlands) equipped with a 96-well plate processor, 4 lasers (405 nm, 488 nm, 561 nm and 638 nm), 48 detectors (16 violet, 14 blue, 10 yellow/green and 8 red) and the specific software SpectroFlo^®^ v.3.3.0 for the analysis of fluorescent signals. Spermatozoa were gated using appropriate regions using FS/SS scatters and discriminating non-sperm events plotting H342/SSC.

##### Sperm Membrane and Acrosome Integrity

Sperm viability was evaluated by staining with propidium iodide (PI, final concentration 16 µM) (Merck KGaA, Darmstadt, Germany), a nuclear dye that penetrates damaged plasma membranes in non-viable spermatozoa. Additionally, double staining with FITC-PNA (final concentration 15 µg/mL) (Merck KGaA) was conducted to simultaneously determine the percentage of spermatozoa that had undergone the acrosome reaction. After incubation at 37 °C in darkness for 15 min, samples were assessed by flow cytometry. The monitored parameters were FS log, SS log, FL2 log (PI) and FL1 log (PNA) and, for the gated sperm cells, percentages of viable (PI-) and acrosome-intact spermatozoa (PNA-) were evaluated.

##### Apoptosis and Mitochondrial Activity

Non-apoptotic-like cells and mitochondrial inner membrane potential were assessed by double staining with Yo-Pro-1 (final concentration 40 nM) and MitoTracker deep red (125 nM final concentration), both purchased from Thermo Fisher Scientific Inc., following incubation at 37 °C for 15 min in darkness. Yo-Pro-1 is a DNA dye that penetrates cells with destabilized membranes, a process that occurs in the early stages of apoptosis, whereas MitoTracker is a carbocyanine-based dye that stains active mitochondria in live cells. 

These techniques were performed as previously described [19,20]. The monitored parameters after analysis by flow cytometry were FS log, SS log, FL1 log (Yo-Pro-1) and FL4 log (MitoTracker) and, for the gated sperm cells, percentages of apoptotic (Yo-Pro-1+) and high-mitochondrial-activity spermatozoa (MitoTracker+) were evaluated.

##### DNA Damage Analysis

DNA fragmentation was assessed by the sperm chromatin structure assay (SCSA) as described by Evenson [21]. Briefly, SCSA evaluates DNA fragmentation and compaction in sperm by using acid denaturation of the sperm DNA and the intercalating fluorochrome acridine orange (AO). AO changes from green to red depending on its binding state, being green when bound to double-stranded DNA (dsDNA) and red when bound to single-stranded DNA (ssDNA) formed by DNA denaturation. Sperm cells from each ejaculate were diluted with TNE buffer (0.01 M Tris-HCl, 0.15 M NaCl and 1 mM disodium EDTA; pH 7.4) to a concentration of 2 × 10^7^ sperm cells/mL. Samples were immediately stored at −20 °C and shipped to the reference laboratory (INDEGSAL, León, Spain) with controlled freezing temperature within 24 h. Upon arrival, samples were stored at −80 °C until processing. Buffer TNE protects DNA from damage during the freezing preservation upon delivery to the external laboratory for SCSA analysis, so −20 and −80 °C storage do not affect DNA fragmentation [22,23].

For analysis, 100 µL aliquots were thawed and treated with 400 µL of acid-detergent solution containing 0.1% Triton X-100, 0.15 mol/L NaCl and 0.08 mol/L HCl (pH 1.2). After 30 s, 1.2 mL of staining buffer (6 µg/mL acridine orange, 100 mmol/L citric acid, 200 mmol/L Na_2_HPO_4_, 1 mmol/L disodium EDTA and 0.15 mol/L NaCl, pH 6.0) was added, and samples were analyzed by flow cytometry within 3 min after AO addition.

The DNA fragmentation index (DFI) reading was calculated as the ratio between the red fluorescence and the total fluorescence (red + green) of individual spermatozoa. Threshold values were established to distinguish between moderate DFI (mDFI; cut-off at 0.25 DFI) and high DFI (hDFI; cut-off at 0.75 DFI) [18,24]. Spermatozoa exceeding these thresholds were classified accordingly. However, both moderate and high DNA fragmentation can lead to fertility issues, making the cut-off values more theoretical than practical. Therefore, for this study, the total DFI (DFI), comprising both mDFI and hDFI, was considered. The sperm nuclear lower compaction, as indicative of sperm immaturity, was assessed by calculating the proportion of spermatozoa with green fluorescence over 65% of the total fluorescence, considered as spermatozoa with high DNA stainability (%HDS).

The presence of PARP1, cPARP and PAR in semen samples was detected using FITC conjugates and flow cytometry. Sperm samples were thawed and 100 µL per sample was dispensed in 96-well dishes, washed with phosphate-buffered saline (PBS, 1000× *g*, 11 min, 4 °C), and then fixed for 20 min in 4% formaldehyde in PBS at room temperature. After fixation, samples were incubated with 10 mM dithiothreitol (DTT) in PBS for 15 min at 37 °C for chromatin disulfide bond reduction. The permeabilization of the spermatozoa was carried out with incubation in 0.1% Triton in 0.1% sodium citrate solution for 30 min on ice. Specific unions were blocked by incubating samples in a 4% bovine serum albumin (BSA) in PBS for 1 h at room temperature. The samples were then incubated overnight at 4 °C with a 1:200 solution in PBS of the primary antibodies anti-PARP1 (436400, Invitrogen, Waltham, MA, USA), anti-cPARP (44–698 G, Invitrogen) and anti-PAR (ab14459, Abcam). The specificity of the primary antibodies was tested by the peptide blocking method. Antibodies were neutralized with the corresponding immunizing blocking peptides: A42573 (Invitrogen) for anti-PARP1; MBS9620327 (MyBioSource) for anti-cPARP; #4336-100-01 (R&D Systems, Bio-Techne, Minneapolis, MN, USA) for anti-PAR antibody. The secondary antibodies used were specifically raised against the primaries and were conjugated to fluorochromes with non-overlapping spectra: Alexa Fluor^®^-647 (ab150111, Abcam), -488 (ab150081, Abcam) and -568 (A11041, Invitrogen), respectively. Finally, sperm samples were incubated with a Hoechst 33342 (H342) solution in PBS at 2.7 µM to stain all cell nuclei and enable detection of all DNA events by flow cytometry. The spermatozoa (gated events) were plotted as PARP1/H342, cPARP/H342 and PAR/H342. Data of at least 5000 spermatozoa were acquired per sample. The parameters registered were the mean fluorescence intensity (MFI) of each fluorochrome PARP1_MFI, cPARP_MFI and PAR_MFI. Data were processed using the Weasel v.3.7 software (Frank Battye, Melbourne, Australia). In addition, the ratios cPARP/PARP1 and PAR/PARP1 were calculated.

### 2.5. Farm Trial: Insemination and Monitoring of Inseminated Sows

Post-cervical artificial insemination (PCAI) sperm doses were transported to two commercial sow farms located in Huesca province (Northeastern Spain). Farm size was 2500–2700 sows, and inseminations were performed from January to April 2022. Sows were heat-checked using a teaser boar and heat signals such us immobility and vulvar changes were registered. Only animals in heat were used for this trial.

A total of 605 sows from DanBred hyperprolific genetics were inseminated (40.1 ± 7.8 females inseminated per boar) with PCAI by using a specific catheter and rod (Magapor SL). Weekly batches of 40 females were inseminated over 9 consecutive weeks with AI doses from group 1, resulting in a total of 354 inseminated sows. Also, weekly batches of about 55 females were inseminated over 6 weeks with semen doses from boars in group 2, resulting in a total of 251 inseminated sows. Each sow was artificially inseminated with the semen from the same male throughout the entire estrus.

Finally, fertility and prolificacy values for each ejaculate, provided by the corresponding multiplier farms, were collected. For each ejaculate, averages from all inseminated sows were calculated, and therefore fertility and prolificacy were evaluated as average ultrasound fertility (%), average farrowing rate (%), average total born and average alive born (%).

### 2.6. Statistical Analysis

IBM SPSS Statistics 26.0 software (IBM Corp., Armonk, NY, USA) was used. The Shapiro–Wilks test was used for assessing the normal distribution of considered variables. In case of non-normal distribution, variables were submitted to adequate transformations (log10, square root, arcsine). Normally distributed variables were summarized as mean and SD (standard deviation). For non-normally distributed variables in spite of transformations (cathepsin B, midpiece, average ultrasound fertility and average farrowing rate fertility), median and interquartile range (IQR) were used. Comparison between groups 1 and 2 was carried out by one-way analysis of variance (ANOVA) for normally distributed variables (original or transformed variables). The Mann–Whitney U test was used for non-normally distributed variables in spite of transformations. Differences were accepted as statistically significant when *p*-values were <0.05.

The large set of variables for sperm characteristics from groups 1 and 2 was separately submitted to principal component analysis (PCA). This statistical technique allows one to reduce a large set of variables into a smaller set (principal components, PCs), accounting for most of the variance in the original variables. Previously, these variables were standardized to avoid possible bias due to the use of different measurement scales [25]. Variables to be considered for the PCA must be linearly related. Hence, variables must show at least one correlation coefficient > 0.3 (absolute value) in the correlation matrix. The Kaiser–Meyer–Olkin index (KMO index) is a linear relationship measurement; only variables with KMO index > 0.5 were considered for the PCA [26]. The number of PCs to be retained was decided based on the eigenvalue-one criterion [27] and the scree plot [28]. Orthogonal rotation (varimax) was applied to these retained components.

Next, to the retained PCs a cluster analysis was applied; seminal doses were grouped based on these PCs. Initially, a hierarchical agglomerative procedure was used to define the number of clusters by the elbow rule based on Ward’s method. Then, the clusters were formed by the k-means procedure [29]; these clusters were groups of seminal doses sharing similar characteristics of quality. Comparison of sperm characteristics among clusters was carried out by one-way ANOVA or, alternatively, Kruskal–Wallis test. Differences were accepted as statistically significant when *p*-values were <0.050. For multiple comparisons, Bonferroni correction was used.

## 3. Results

Table 2 shows the characteristics of sperm from boars in both groups 1 and 2. β-galactosidase was studied only in group 1; due to technical problems, these data are not available in group 2. No normal distribution was detected for 18 sperm quality variables (Shapiro–Wilks test; *p* < 0.05). Therefore, log10 transformation was used for fructose concentration, SOD, β-galactosidase, GPX5, AF, damaged acrosomes, early apoptosis, DFI and HDS. Square root transformation was applied to protein and citrate concentrations, tail and PD. Acro, sHOST, sORT, viability and mitochondrial activity were transformed by arcsine function. No transformation could normalize the distributions of cathepsin B, head and midpiece; therefore, median and interquartile range (IQR) were used for resuming these variables. Significant differences between groups 1 and 2 were detected for 24/43 (55.8%) comparisons of sperm quality variables (*p* < 0.05).

However, average ultrasound fertility and average farrowing rate (%) could not be normalized by any transformation; median and IQR are shown in Table 2. Average farrowing rate (%) and average alive born (%) significantly differed between groups 1 and 2 (*p* < 0.05, see Table 2). For average farrowing rate, both groups showed an equal median (100%), but the IQR was very different in each group: while in group 1 the different values were all very close to 100%, in group 2 they varied widely from each other, and the statistical test shows this clearly.

These results led us to consider groups 1 and 2 separately in the subsequent analyses. For PCA, only variables with adequate valid data were initially considered, excluding β- galactosidase (group 1: *n* ≥ 79, 40 variables; group 2: *n* ≥ 20, 41 variables). Finally, only 14/40 (35.0%) and 15/41 (36.6%) variables met the conditions for PCA in groups 1 and 2, respectively. Based on the criteria explained in the statistical methodology, PCA used the information for these original variables (14 and 15 for group 1 and 2, respectively) in only three PCs for both groups 1 and 2 (PC1, PC2 and PC3) and, depending on the original variables they included, we named them speed (PC1), linear path (PC2) and DNA damage (PC3). In group 1, they explained 53.6%, 18.0% and 12.2% of total variance, respectively. Slightly different percentages were found in group 2 (43.1%, 27.9% and 19.2%, respectively). The cumulative percentage of variance explained by them was 83.9% and 90.2% for group 1 and 2, respectively.

The correlation coefficients for each original variable and the estimated PCs (rotated components coefficients, loadings) are shown in Table 3, where coefficients below │0.3│ (absolute value) were excluded for clarity. Also, Table 3 shows the communalities for every original variable (proportion of each original variable’s variance that is accounted for by PCA).

Positive correlation values mean that the original variable and PC move in the same direction, while negative correlation values indicate that they move in opposite directions. In addition, when an original variable has a strong effect on a PC, the correlation shows a large absolute value.

For PC1, results about strongly correlated variables were similar for both groups. In group 1, PC1 loads very strongly on fast spermatozoa (%), VCL, ALH, intermediate spermatozoa (%), VAP, static spermatozoa (%), TMot (%) and slow spermatozoa (%). In group 2, PC1 also loads strongly on fast spermatozoa (%), VCL, ALH, intermediate spermatozoa (%), VAP, static spermatozoa (%) and TMot (%), but no significant correlation was found for slow spermatozoa (%). However, variables with low correlations for PC1 were different for the considered groups.

Regarding PC2, results about strongly correlated variables were also similar for both groups. High correlation values were found for PMot, STR, VSL and LIN in both group 1 and group 2. However, PC2 included BCF only in group 2. Additionally, these original variables also have correlation values higher than │0.3│with PC1, especially in group 1; therefore, no simple structure was found for PC1 and 2.

Original variants included in PC3 differ between groups 1 and 2 (see Table 3), but cPARP/PARP1 and PAR/PARP1 (PC3, group 1) are clearly related to cPARP, PARP1 and PAR (PC3, group 2).

Based on these three PC, the seminal doses sharing similar characteristics were grouped into four groups (clusters) from each group considered. Comparisons of sperm, fertility and prolificacy characteristics among clusters from groups 1 and 2 are shown in Table 4 and Table 5, respectively.

As can be seen in Table 4, from group 1, cluster 1 is characterized by low values in PC1, PC2 and PC3; cluster 2 shows high values in both PC1 and PC2 but low values in PC3; cluster 3 presents intermediate value in PC1, high value in PC2 and the highest value in PC3; finally, cluster 4 exhibits high value in PC1, lowest value in PC2 and intermediate value in PC3. Table 4 also shows similar tendencies in original variables included in these three PCs in the four clusters from group 1. No significant differences among clusters were found for fertility and prolificacy characteristics.

The results in Table 5 show that in group 2, cluster 1 shows lowest values in PC1, low values in PC2 and high values in PC3; cluster 2 presents highest values in PC1, low values in PC2 and high values in PC3; cluster 3 was characterized by intermediate values in PC1, highest values in PC2 and intermediate values in PC3; finally, cluster 4 exhibits high values in PC1, low values in PC2 and lowest values in PC3. Similar tendencies in original variables included in these three PCs were found in these clusters. These clusters did not differ significantly for fertility and prolificacy characteristics.

Differences among clusters for variables not included in the PC analysis were also explored. Table 6 shows significant differences among clusters in group 1 (*p* < 0.050). Significant difference was found for BCF (*p* = 0.044); however, multiple comparisons failed to detect significant differences between specific clusters, even though cluster 4 showed the lowest mean. Also, clusters differed significantly for PARP (*p* < 0.001); clusters 1 and 2 showed similar highest values, which differed from both cluster 3 (lowest value) and cluster 4 (intermediate value). PARP1 was not included in any PC, but this variable was involved in the calculation of both cPARP/PARP1 and PAR/PARP1 (PC3).

In group 2, four variables not included in the obtained PC analysis significantly differed among clusters (see Table 7). For cathepsin B (*p* = 0.013), cluster 3 (lowest value) showed significant differences with respect to cluster 4 (highest value), while clusters 1 and 2 (intermediate value) did not differ from any other cluster. Significant differences among clusters also were found for AF (*p* = 0.012): clusters 1 and 4 did not differ between themselves or with the other clusters, while extreme values were found in clusters 2 and 3. Mitochondrial activity differed among clusters (*p* = 0.046); cluster 1 presented the highest values and cluster 3 the lowest ones, clusters 2 and 4 being intermediate between the previous ones. Finally, significant differences among clusters were observed for HDS (*p* = 0.020); the highest values occurred in both clusters 2 and 4 and the lowest value corresponded to cluster 3, with cluster 1 being intermediate.

## 4. Discussion

In this work, as said before, boars from different genetic lines were studied in different periods of the year. No study was conducted to detect possible differences in semen motility between different months. Some authors claim that sperm motility in boars is affected by the period of the year [30], while others conclude that sperm quality parameters (including total and progressive motility) are affected neither by photoperiod nor temperatures in boars housed in environmentally controlled buildings [31].

However, the data from both genetic lines coming from two different periods of the year were studied separately, so that possible biases due to this difference were avoided as much as possible. On the other hand, the results from PCA and clustering were quite similar in both groups, and in both cases no relationship of clustering with reproductive performance was found.

Several previous studies considered sub-population of spermatozoa in boars [32,33], while others [34] preferred to study the different ejaculates as a whole, because the spermatozoa in the ejaculate responsible for the fertilization of the sow is unknown, as well as the sub-population to which it belongs. In cattle, fertility is known to vary among sires and even among ejaculates from the same sire [11]. In the present study, we worked on ejaculates following this idea.

The order of PCs is not arbitrary; the first component will explain the greatest amount of total variance, with each subsequent component accounting for relatively less of the total variance [35]. In a similar study in bulls, the first principal component was related to sperm velocity, while the second component was related to the linearity of sperm movement; the third component, which was detected only before freezing, was related to BCF [11]. Our results would resemble these ones in terms of the relative importance of our first two PC.

Barquero et al. [9] detected two PCs from CASA kinematics variables in Pietrain and Duroc × Pietrain ejaculates. The first PC (linear trajectory) was responsible for 53% of the variance and was associated with LIN, STR, WOB and VSL, while the second PC (velocity; 47% of the variance) was related with VSL, VCL, ALH and VAP; BCF showed an eigenvector <0.6. These results suggested a relatively larger effect of sperm linear trajectory on total variance than that of velocity.

Our results showed great similarity between groups 1 and 2, although the data come from different genetic lines of the Pietrain breed. Apart from WOB, our results were similar, although LIN, STR and VSL were associated with PC2 (linear path) and VSL, VCL, ALH and VAP with PC1 (speed); also, VSL was associated with both PC1 and PC2. In addition, in group 2, BCF showed a correlation high enough to be considered in PC2 (linear path). However, our results for PCs suggested that sperm speed had a greater effect on the total variance than the sperm linear path, the effect of intact DNA being even smaller. This difference with the earlier study [19] would be due to the fact that we have considered more speed variables and DNA damage markers; therefore, the percentage of the total variance explained by PC1 and PC2 varied.

Cluster analysis captures the natural structure of data and, therefore, divides them into meaningful groups [36]. In the present work, these clusters are useful for data summarization based on the large amount of information obtained from semen analysis. However, no differences among clusters were found for fertility or prolificacy. Fertility of a boar must be assessed on a large number of sows for a long time, and during this period, its fertility could change [37]. On the other hand, sow-related factors such as housing [38], weaning–estrus interval [39], hormonal activity [40] and lifetime performance [41] also influence both fertility and prolificacy.

The relationships between sperm parameters and fertility/prolificacy are controversial and require important qualifications. Many events are implicated in the fertilization process and sperm tests only evaluate a small proportion of them; therefore, the found relationships usually are of little value [42]. Tsakmakidis et al. [43] showed that boar fertility can be predicted from sperm morphology and chromatin integrity, while the number of live piglets per parturition was not correlated to these characteristics. Barquero et al. [34] did not find significant differences between ejaculate clusters based on kinematic characteristics in fertility variables; also, these clusters did not show predictive capacity for litter size variables. Michos et al. [44] concluded in favor of the usefulness of immotile sperm in estimating the fertility of boars in the field, but also the important role played by membrane biochemical activity, mitochondrial membrane potential, osteopontin and glutathione peroxidase 5. Current sperm analysis effectively detects ejaculates with very poor quality; these ejaculates are considered as associated with poor fertility and eliminated [42]. Percentage of normal sperm has significantly influenced both numbers of pigs born alive and litter size; also, abnormal sperm head morphology and retained distal cytoplasmic droplets have had a significant effect on litter size [45]. Efficient quality control is currently underway [46,47] and, once the minimum requirements have been met, no relationships with fertility parameters would be expected. Furthermore, other characteristics not usually considered in sperm analysis play a role in fertility and prolificacy. Bacterial diversity and profile of ejaculated boar semen vary in different seasons and are related to fertility capacity [48]. Differences were found in DNA methylation when comparing boars of high and low fertility, with differentially methylated regions (DMRs) observed more frequently in highly fertile individuals [49].

In group 1, BCF and PARP1_MFI do not provide any additional relevant information to that provided by the three PCs. However, in group 2, cathepsin B, AF, mitochondrial activity and HDS differed significantly among clusters. High concentrations of cathepsin B in boar seminal plasma are associated with reduced TMot and PMot and low sperm morphology [50]. Boar abnormal forms showed significant predictive capacity for litter size [34]. Functional mitochondria are an essential marker of fertilization capacity [51]. Diagnosis of DNA damage contributes to a better understanding of male infertility [52]. Unfortunately, the small number of ejaculates included in each cluster of group 2 prevents drawing well-founded conclusions.

## 5. Conclusions

PCA showed similar results based on kinetics, flow cytometric characteristics and degree of DNA damage in ejaculates from two Pietrain genetic lines. Also, ejaculates were classified into four clusters in both lines. Therefore, PCA and clustering achieved adequate description of these ejaculates, although no differences were found among clusters for fertility or prolificacy. Due to efficient quality control of semen, no relationships with fertility parameters would be expected once the minimum requirements have been met.

## Figures and Tables

**Table 1:** Limits for ejaculates of adequate seminal quality.

Variable	Limit for Acceptance
Total motility (Tmot)	>70%
Progressive motility (Pmot)	>50%
Viability	>80%
Acrosomal damage	<20%
Mitochondrial activity	>70%
Early apoptosis	<14%
Abnormal forms (AF)	≤25–30%
Head	<5%
Midpiece	<5%
Tail	<10%
Proximal drop (PD)	<15%
Distal drop (DD)	<15%
Short hypo-osmotic swelling test (sHOST)	>50%
Short osmotic resistance test (sORT)	>70–75%

**Table 2:** Characteristics of ejaculates from boars in groups 1 and 2.

Variable	Group 1		Group 2		*p*-Value
	*n*	Mean ± SD; Median [IQR]	*n*	Mean ± SD; Median [IQR]	
Protein (mg/mL)	90	6.7386 ± 1.31267	28	4.7594 ± 1.23925	<0.001
Citrate (mg/dL)	90	12.4196 ± 2.71401	29	12.8822 ± 4.20687	0.491
Fructose (log10 mg/dL)	87	0.6600 ± 0.19237	26	0.4360 ± 0.15004	<0.001
Zinc (µg/dL)	90	4510.0189 ± 1594.17875	26	3750.1846 ± 1053.74954	0.024
SOD (log10 U/mL)	87	0.2266 ± 0.24489	25	0.0056 ± 0.16432	<0.001
Beta-Galactosidase (log10 nmol/min/mg)	89	18.3396 ± 29.57560		NA	
GPX5 (log10 ng/mL)	44	2.2383 ± 0.14754	9	1.8744 ± 0.03192	<0.001
Cathepsin B (RFU)	49	21,227.00 [1061]	27	19943.00 [722]	<0.001
ALP(U/mL)	20	5.6702 ± 2.53767	2	1.3316 ± 1.67178	0.030
pH	79	7.4334 ± 0.07188	29	7.4283 ± 0.09769	0.776
AF (log10%)	89	1.2926 ± 0.18749	29	1.0724 ± 0.14054	<0.001
Head (%)	89	1.00 [2]	29	1.00 [1]	0.243
Tail (%)	89	3.1337 ± 1.13333	29	1.4256 ± 0.68312	<0.001
Midpiece (%)	90	0.00 [1]	29	0.00 [1]	0.826
PD (%)	89	2.0195 ± 0.92947	29	2.0172 ± 0.53441	0.990
DD (%)	89	4.2135 ± 2.81017	29	4.6552 ± 2.36456	0.447
Acro (arsin %)	89	1.1780 ± 0.08508	29	1.1422 ± 0.22374	0.209
TMot (%)	89	90.3119 ± 5.60284	29	92.8220 ± 2.98770	0.023
PMot (%)	89	64.8374 ± 6.85426	29	63.4036 ± 11.70502	0.420
Fast spermatozoa (%)	89	66.0303 ± 13.51672	29	61.7668 ± 16.95567	0.169
Intermediate spermatozoa (%)	89	20.8340 ± 6.89902	29	26.3456 ± 11.78001	0.003
Slow spermatozoa (%)	89	3.4475 ± 2.67707	29	4.7095 ± 3.98397	0.055
Static spermatozoa (%)	89	96.881 ± 5.60284	29	7.1780 ± 2.98770	0.023
VCL (µm/s)	89	67.8945 ± 9.44221	29	67.6162 ± 13.60399	0.902
VSL (µm/s)	89	30.4954 ± 3.53074	29	31.0644 ± 8.56279	0.611
VAP (µm/s)	89	46.9815 ± 6.88823	29	48.8559 ± 11.14385	0.283
LIN (%)	89	45.3794 ± 5.52790	29	45.7437 ± 8.50739	0.790
STR (%)	89	65.5962 ± 7.52125	29	63.4163 ± 9.20783	0.203
WOB (%)	89	69.2312 ± 3.61757	29	71.9234 ± 5.16977	0.002
ALH (µm)	89	2.5119 ± 0.27532	29	2.4237 ± 0.29651	0.144
BCF (Hz)	89	6.5153 ± 0.32342	29	6.7763 ± 0.54329	0.002
sHOST (arsin%)	89	0.8045 ± 0.12578	29	0.8838 ± 0.08634	0.002
sORT (arsin%)	89	0.8603 ± 0.23835	29	0.9895 ± 0.10674	0.006
Viability (arsin%)	89	1.1743 ± 0.10778	29	1.0916 ± 0.16653	0.002
Damaged acrosomes (log10%)	89	0.9603 ± 0.29151	29	1.1466 ± 0.17580	0.002
Mitochondrial activity (arsin %)	89	1.1538 ± 0.11171	29	1.1354 ± 0.06828	0.404
Early apoptosis (log10%)	89	1.0083 ± 0.18103	29	0.7977 ± 0.15423	<0.001
cPARP_MFI	88	11.4181 ± 0.24094	28	11.8417 ± 0.31458	<0.001
PARP1_MFI	88	12.1382 ± 0.32742	28	12.3613 ± 0.35230	0.003
PAR_MFI	88	12.5525 ± 0.41542	28	12.8826 ± 0.42295	<0.001
cPAR/PARP	88	0.9413 ± 0.02899	28	0.9581 ± 0.01379	0.004
PAR/PARP1	88	1.0346 ± 0.03594	28	1.0422 ± 0.01702	0.282
DFI (log10%)	88	0.5442 ± 0.3340	28	0.3185 ± 0.50280	0.007
HDS log10(%)	88	0.8840 ± 0.09164	28	0.8538 ± 0.10143	0.141
Average ultrasound fertility (%)	74	100.000 [0.0]	29	100.000 [7.7]	0.794
Average farrowing rate (%)	74	100.000 [0.0]	29	100.000 [13.4]	0.049
Average total born	74	20.4052 ± 2.22923	29	19.8421 ± 1.64295	0.22
Average alive born (%)	74	89.7984 ± 4.67538	29	83.6526 ± 5.12710	<0.001

Values are mean ± SD (standard deviation) or, alternatively, median and interquartile rank [IQR, in brackets] for cathepsin B, head, midpiece, average ultrasound fertility and average farrowing rate. NA: not analyzed.

**Table 3:** Rotated component matrix: loadings of principal components 1, 2 and 3 related to original variables and communalities for every original.

Variable	Group 1				Group 2			
	Rotated Component Coefficients			Communalities	Rotated Component Coefficients			Communalities
	1	2	3		1	2	3	
TMot	0.873			0.830	0.819			0.815
PMot		0.798		0.796	0.376	0.852		0.915
Fast spermatozoa	0.979			0.976	0.942			0.970
Intermediate spermatozoa	−0.896			0.822	−0.952			0.961
Static spermatozoa	−0.873			0.830	−0.819			0.815
Slow spermatozoa	−0.812			0.735				
VCL	0.958			0.920	0.980			0.970
ALH	0.897			0.808	0.840			0.800
VAP	0.876			0.771	0.940			0.918
STR	−0.573	0.788		0.950		0.936		0.902
VSL	0.523	0.765		0. 859	0.679	0.683		0.956
LIN	−0.613	0.754		0.944		0.982		0.979
BCF					0.316	0.784		0.727
PARP1_MFI							0.943	0.908
cPARP_MFI							0.946	0.947
PAR_MFI							0.940	0.960
cPAR/PARP			0.864	0.760				
PAR/PARP1			0.850	0.751				

Coefficients below │0.3│ (absolute value) were excluded for clarity.

**Table 4:** Characteristics of clusters from group 1 for variables included in the obtained three principal components.

Variable	Cluster 1; *n* = 11 §	Cluster 2; *n* = 21 ¶	Cluster 3; *n* = 28 φ	Cluster 4; *n* = 27 Δ	*p*-Value
	Mean ± SD; Median [IQR]	Mean ± SD; Median [IQR]	Mean ± SD; Median [IQR]	Mean ± SD; Median [IQR]	
PC1	−1.4085 ^a^ ± 0.71234	0.3321 ^b^ ± 0.86504	−0.3164 ^c^ ± 0.79757	0.6437 ^b^ ± 0.64010	<0.001
PC2	−0.4815 ^a^ ± 0.83894	0.8841 ^b^ ± 0.44304	0.4749 ^b^ ± 0.63594	−0.9840 ^a^ ± 0.69876	<0.001
PC3	−0.7976 ^a^ ± 0.66988	−0.8725 ^a^ ± 0.71878	0.9571 ^b^ ± 0.55749	0.0110 ^c^ ± 0.73767	<0.001
Fast spermatozoa (%)	45.1848 ^a^ ± 8.85700	69.7467 ^b,c^ ± 12.39397	63.6803 ^b^ ± 11.75661	73.8115 ^c^ ± 7.85655	<0.001
VCL (µm/s)	55.9000 ^a^ ± 4.99424	70.4094 ^b^ ± 7.84229	64.1001 ^c^ ± 6.58479	74.2147 ^b^ ± 8.00436	<0.001
ALH (µm)	2.1943 ^a^ ± 0.19278	2.6018 ^b^ ± 0.24798	2.4063 ^a^ ± 0.19504	2.6785 ^b^ ± 0.25136	<0.001
Intermediate spermatozoa (%)	29.2699 ^a^ ± 3.72531	18.1685 ^b^ ± 6.43821	23.1776 ^c^ ± 6.12216	17.2141 ^b^ ± 5.41188	<0.001
VAP (µm/s)	39.3966 ^a^ ± 3.37743	48.1549 ^b,c^ ± 5.34746	44.3685 ^a,b^ ± 5.25757	51.2188 ^c^ ± 6.55598	<0.001
Static spermatozoa (%)	17.0925 ^a^ ± 6.62353	9.6535 ^b^ ± 5.2176	9.9307 ^b^ ± 5.1019	6.5149 ^b^ ± 2.7595	<0.001
TMot (%)	82.9075 ^a^ ± 6.62353	90.3465 ^b^ ± 5.21763	90.0693 ^b^ ± 5.10190	93.4851 ^b^ ± 2.75953	<0.001
Slow spermatozoa (%)	8.4528 ^a^ ± 3.39256	2.4312 ^b^ ± 1.64620	3.2113 ^b^ ± 1.79818	2.4594 ^b^ ± 1.30086	<0.001
TMot (%)	57.7544 ^a^ ± 5.40700	69.1318 ^b^ ± 3.82054	68.9610 ^b^ ± 4.58456	60.0093 ^a^ ± 5.23735	<0.001
STR (%)	68.5058 ^a^ ± 6.60440	69.2856 ^a^ ± 4.18707	69.9551 ^a^ ± 3.98705	57.1605 ^b^ ± 5.03353	<0.001
VSL (µm/s)	26.8989 ^a^ ± 2.59480	33.2147 ^b^ ± 2.72184	30.9410 ^c^ ± 3.16079	29.081 ^a,c^ ± 2.89799	<0.001
LIN (%)	48.3921 ^a^ ± 5.72870	47.4346 ^a^ ± 3.61200	48.37503 ^a^ ± 3.26459	39.3185 ^b^ ± 3.03620	<0.001
cPARP/PARP	0.9173 ^a^ ± 0.02122	0.9192 ^a^ ± 0.02634	0.9639 ^b^ ± 0.01773	0.9443 ^c^ ± 0.02399	<0.001
PAR/PARP1	1.0084 ^a^ ± 0.02507	1.0125 ^a^ ± 0.02385	1.0637 ^b^ ± 0.03128	1.0297 ^a^ ± 0.02399	<0.001
Average ultrasound fertility (%)	100.000 [3.1]	100.000 [4.2]	100.000 [0.0]	100.000 [0.0]	0.938
Average farrowing rate (%)	100.000 [6.3]	100.000 [0.0]	100.000 [0.0]	100.000 [0.0]	0.934
Average total born	20.7833 ± 1.99389	20.4311 ± 2.20974	19.7485 ± 1.96845	20.6199 ± 2.49045	0.487
Average alive born (%)	87.8262 ± 4.36007	89.8729 ± 4.85416	90.1484 ± 5.31532	89.8835 ± 4.11972	0.607

Values are mean ± SD (standard deviation) or, alternatively, median and interquartile rank [IQR, in brackets] for average ultrasound fertility and average farrowing rate. Different superscript letters (a, b, c) on the same line indicate significant differences (*p* < 0.050). For average ultrasound fertility, average farrowing rate, average total born and average alive born, lower *n* values were available: § *n* = 10; ¶ *n* = 14; φ *n* = 23; Δ *n* = 25.

**Table 5:** Characteristics of clusters from group 2 for variables included in the obtained three principal components.

Variable	Cluster 1 (*n* = 7)	Cluster 2 (*n* = 13)	Cluster 3 (*n* = 3)	Cluster 4 (*n* = 5)	*p*-Value
	Mean ± SD; Median [IQR]	Mean ± SD; Median [IQR]	Mean ± SD; Median [IQR]	Mean ± SD; Median [IQR]	
PC1	−1.4095 ^a^ ± 0.57800	0.7909 ^b^ ± 0.44732	−0.0151 ^b,c^ ± 0.04783	−0.0741 ^c^ ± 0.36106	<0.001
PC2	−0.3389 ^a^ ± 0.80736	−0.2709 ^a^ ± 0.73542	1.9090 ^b^ ± 0.86050	0.0336 ^a^ ± 0.77792	0.001
PC3	0.4293 ^a^ ± 0.39941	0.4309 ^a^ ± 0.71028	−0.1383 ^a^ ± 0.12647	−1.6385 ^b^ ± 0.83228	0.001
Fast spermatozoa (%)	38.9605 ^a^ ± 12.18404	72.3066 ^b^ ± 8.19884	72.3959 ^b^ ± 3.61912	64.8997 ^b^ ± 7.61626	<0.001
VCL (µm/s)	49.5010 ^a^ ± 6.96756	78.1423 ^b^ ± 6.06587	67.7180 ^b^ ± 2.85053	69.8007 ^b^ ± 7.40771	<0.001
ALH (µm)	2.1192 ^a^ ± 0.22886	2.6272 ^c^ ± 0.16290	2.2159 ^a,b^ ± 0.22084	2.5079 ^b,c^ ± 0.26523	<0.001
Intermediate spermatozoa (%)	42.2119 ^a^ ± 6.78987	18.1467 ^b^ ± 4.50306	20.6377 ^b^ ± 4.04192	24.9876 ^b^ ± 7.28262	<0.001
VAP (µm/s)	33.5237 ^a^ ± 4.61205	56.8409 ^b^ ± 4.76099	53.7685 ^b^ ± 4.40941	50.4416 ^b^ ± 5.30836	<0.001
Static spermatozoa (%)	10.9849 ^a^ ± 2.97889	5.5581 ^b^ ± 1.22475	4.6091 ^b^ ± 1.86427	6.8986 ^b^ ± 0.95353	<0.001
TMot (%)	89.0151 ^a^ ± 2.97889	94.4419 ^b^ ± 1.22475	95.3909 ^b^ ± 1.86427	93.1014 ^b^ ± 0.95353	<0.001
PMot (%)	54.7169 ^a^ ± 13.20829	63.6333 ^a,b^ ± 7.49602	80.4518 ^b^ ± 4.36665	67.1436 ^a,b^ ± 10.74330	0.006
STR (%)	61.9127 ^a^ ± 8.34311	60.2179 ^a^ ± 7.88531	78.6689 ^b^ ± 6.41470	65.6590 ^a,b^ ± 7.62090	0.010
VSL (µm/s)	20.8002 ^a^ ± 4.02471	34.1133 ^b^ ± 4.29268	42.4527 ^b^ ± 6.75166	33.4083 ^b^ ± 6.74143	<0.001
LIN (%)	41.9743 ^a^ ± 5.73132	43.8142 ^a^ ± 5.87890	62.6168 ^b^ ± 8.72155	47.5575 ^a^ ± 6.64438	<0.001
BCF (Hz)	6.3307 ^a^ ± 0.39880	6.7958 ^a^ ± 0.41068	7.6763 ^b^ ± 0.46764	6.9679 ^a,b^ ± 0.23032	<0.001
cPARP_MFI	12.0728 ^a^ ± 0.11615	11.9372 ^a^ ± 0.23737	11.7066 ^a,b^ ± 0.09060	11.3512 ^b^ ± 0.19266	<0.001
PARP1_MFI	12.5320 ^a^ ± 0.14422	12.5168 ^a^ ± 0.26409	12.1667 ^a,b^ ± 0.12494	11.8345 ^b^ ± 0.29098	<0.001
PAR_MFI	13.2006 ^a^ ± 0.09357	13.0084 ^a^ ± 0.28830	12.7178 ^a,b^ ± 0.02211	12.2090 ^b^ ± 0.35881	<0.001
Average ultrasound fertility (%)	100.000 [0.0]	100.000 [9.2]	100.000 [-]	100.000 [5.0]	0.673
Average farrowing rate (%)	100.000 [11.1]	100.000 [14.8]	87.500 [-]	100.000 [5.6]	0.214
Average total born	19.1309 ± 1.18501	20.0865 ± 1.83732	18.6190 ± 0.85846	20.5971 ± 1.63092	0.236
Average alive born (%)	84.8822 ± 5.20470	82.8528 ± 4.16278	81.6802 ± 12.14253	84.6389 ± 2.62393	0.750

Values are mean ± SD (standard deviation) or, alternatively, median and interquartile rank [IQR, in brackets] for average ultrasound fertility and average farrowing rate. Different superscript letters (a, b, c) on the same line indicate significant differences (*p* < 0.050). [-]: IQR not estimated.

**Table 6:** Significant differences among clusters from group 1 for variables not included in the obtained three principal components.

Variable	Cluster 1 (*n* = 11)	Cluster 2 (*n* = 21)	Cluster 3 (*n* = 28)	Cluster 4 (*n* = 27)	*p*-Value
	Mean ± SD	Mean ± SD	Mean ± SD	Mean ± SD	
BCF (Hz)	6.61667 ± 0.40660	6.6170 ± 0.28659	6.5167 ± 0.29591	6.3785 ± 0.31098	0.044
PARP1_MFI	12.4200 ^a^ ± 0.32185	12.3439 ^a^ ± 0.25806	11.8881 ^b^ ± 0.26731	12.1229 ^c^ ± 0.24098	<0.001

Values are mean ± SD (standard deviation). Different superscript letters (a, b, c) on the same line indicate significant differences (*p* < 0.050).

**Table 7:** Significant differences among clusters from group 2 for variables not included in the obtained three principal components.

Variable	Cluster 1; *n* = 7	Cluster 2; *n* = 13	Cluster 3; *n* = 3	Cluster 4; *n* = 5 §	*p*-Value
	Mean ± SD; Median [IQR]	Mean ± SD; Median [IQR]	Mean ± SD; Median [IQR]	Mean ± SD; Median [IQR]	
Cathepsin B (RFU)	20,176.00 ^a,b^ [1468]	19,638.00 ^a,b^ [684]	19,246.00 ^a^ [-]	20,426.00 ^b^ [-]	0.013
AF (log10%)	1.0850 ^a,b^ ± 0.12044	1.1207 ^a^ ± 0.10240	0.8521 ^b^ ± 0.20060	1.0248 ^a,b^ ± 0.097140	0.012
Mitochondrial activity (arsin%)	1.1728 ^a^ ± 0.04894	1.1451 ^a,b^ ± 0.06156	1.0472 ^b^ ± 0.10914	1.1168 ^a,b^ ± 0.04925	0.046
HDS log10 (%)	0.8403 ^a,b^ ± 0.08349	0.8835 ^a^ ± 0.06418	0.6978 ^b^ ± 0.19773	0.8889 ^a^ ± 0.064000	0.020

Values are mean ± SD (standard deviation) or, alternatively, median and interquartile rank [IQR, in brackets] for cathepsin B. Different superscript letters (a, b) on the same line indicate significant differences (*p* < 0.050). For cathepsin B in cluster 4, lower *n* value was available: § *n* = 3. [-]: IQR not estimated.

## Data Availability

The data are contained within this article and Supplementary Materials.

## Supplementary Materials

The following supporting information can be downloaded at: https://www.mdpi.com/article/10.3390/vetsci11090420/s1, Table S1: List of acronyms.
