# Supplementary material for: Spermiogram, Kinetics, Flow Cytometric Characteristics and DNA Damage Degree in Boar Ejaculates: Summarization and Clustering
Source: Vet Sci. 2024 Sep 9;11(9):420. doi: 10.3390/vetsci11090420 (PMC11435697; doi:10.3390/vetsci11090420)
Supplement: Supplementary file 1 [file vetsci-11-00420-s001.zip › vetsci-3144415-supplementary.pdf]

Table S1: List of acronyms

**Acronym**	**Explanation**
DNA	Deoxyribonucleic Acid
CASA	Computer-Assisted Semen Analysis
PCA	Principal Component Analysis
PC´s	Principal Components
HOST	Hypoosmotic swelling test
ORT	Osmotic resistance test
PARP	Poly (ADP-ribose) polymerase
PAR	Poly (ADP-ribose)
cPARP	Cleaved Poly (ADP-ribose) polymerase
SP	Seminal plasma
SOD	Superoxide dismutase
GPX5	Glutathione peroxidase 5
TMot	Total sperm motility
PMot	Progressive sperm motility
VCL	Curvilinear velocity
VSL	Straight line velocity
VAP	Average velocity path
LIN	Linearity
STR	Straightness
WOB	Wobble
ALH	Amplitude of lateral head displacement
BCF	Beat cross frequency
PD	Proximal droplet
DD	Distal droplet
AF	Abnormal forms
sHOST	Short hypoosmotic twelling test
sORT	Short osmotic resistance test
BTS	Beltsville Thawing Solution
EDTA	Disodium ethylenediamine tetraacetate
Acro %	% of intact acrosomes
PI	Propidium iodide
FITC-PNA	Fluorescein isothiocyanate-Arachis hypogaea lectin
SCSA	Sperm Chromatin Structure Assay
AO	Acridine orange
dsDNA	Double-stranded DNA
ssDNA	Single-stranded DNA
DFI	DNA Fragmentation Index
mDFI	Moderate DNA Fragmentation Index
hDFI	High DNA Fragmentation Index
HDS	High DNA stainability
PBS	Phosphate buffered saline solution

DTT	Dithiothreitol
BSA	Bovine serum albumin
MFI	Mean fluorescence intensity
PCAI	Post-cervical artificial insemination
SD	Standard deviation
IQR	Interquartile range
Anova	Analysis of Variance
KMO	Kaiser–Meyer–Olkin index
ALP	Alkaline phospatase
pH	Potential of hydrogen
DMRs	Differentially Methylated Regions
